# Psychometric properties of the Japanese version of the EQ-5D-Y by self-report and proxy-report: reliability and construct validity

**DOI:** 10.1007/s11136-019-02238-1

**Published:** 2019-06-26

**Authors:** T. Shiroiwa, T. Fukuda, K. Shimozuma

**Affiliations:** 1 Center for Outcomes Research and Economic Evaluation for Health (C2H), National Institute of Public Health, 2-3-6 Minami, Wako, Saitama 351-0197 Japan; 2 Department of Biomedical Sciences, College of Life Sciences, Ritsumeikan University, 1-1-1 Noji-higashi, Kusatsu, Shiga 525-8577 Japan

**Keywords:** EQ-5D-Y, Reliability, Construct validity, General population, PedsQL, Proxy

## Abstract

**Purpose:**

This study aimed to assess psychometric properties of the Japanese version of the EQ-5D-Y (3 levels) with a focus on feasibility, reliability, and construct validity.

**Methods:**

Respondents were recruited from the general populations of three cities in Japan. First, children and adolescents responded to the EQ-5D-Y and PedsQL by self-report. Parents were also asked to evaluate the health states of their children/adolescents using proxy versions of these questionnaires. Next, the EQ-5D-Y was mailed to their residence approximately 2 weeks later, and both children/adolescents and their parents responded to the questionnaire. Reliability was confirmed by self-report test–retest methods and a comparison of self-report responses with proxy responses. Spearman’s correlation coefficients were calculated between responses to the EQ-5D-Y and both responses to and scores of the PedsQL in order to assess construct validity.

**Results:**

A total of 654 children/adolescents from aged 8 to 15 (median age: 11) responded to the questionnaires at both the first- and second-stage surveys. Test–retest agreement was sufficiently high and was influenced by age. Proxy test–retest results revealed that parents’ responses were more reliable compared to the self-report results. Some correlations (|*r*| > 0.3) between items of the EQ-5D-Y and PedsQL were found. Meanwhile, no correlations were found between proxy responses to the EQ-5D-Y and self-report responses to the PedsQL.

**Conclusions:**

The EQ-5D-Y demonstrates reliability and validity among children/adolescents and their parents in Japan. Construct validity of the EQ-5D-Y by self-report was confirmed through comparisons with the PedsQL. Proxy responses to the EQ-5D-Y were more reliable compared to the self-report results, but construct validity was not confirmed in the proxy version.

## Introduction

Measurement of health-related quality of life (HRQOL) of children and adolescents is becoming increasingly important for the evaluation of healthcare technologies. The EuroQol Five-Dimensional Questionnaire, Youth Version (EQ-5D-Y) [1], is designed to be a preference-based measure (PBM) that can be used to calculate quality-adjusted life years (QALYs). Some PBMs, including EQ-5D [2–7], Health Utilities Index (HUI) 2/3 [8–11], and Short Form 6 Dimension (SF-6D) [12–15], have been developed for adults, but only a few measures have been designed specifically for children/adolescents, and include the HUI2 and Child Health Utility-9D [16, 17]. Applying PBMs designed for adults to children can be problematic since the vocabulary may not be child-friendly and thus difficult to understand. In Japanese, both *kanji* (Chinese characters) and *hiragana* (Japanese characters) are used in sentences, and the Japanese version of the EQ-5D-5L uses both. However, since most young children cannot read or understand all *kanji* characters used in the questionnaire, developing PBMs tailored to children/adolescents is necessary in order to evaluate accurately their health states by self-completion (self-report).

HRQOL measures for children/adolescents can also be problematic if the intended meaning of the questions is difficult to understand such that a proper response is prevented. For this reason, HRQOL measures often limit their target population by age or recommend to use proxy-report. [18] The EQ-5D-Y was developed for self-report by children/adolescents aged 8–15. For children aged 4–7, a proxy version of EQ-5D-Y can be used [19]. Other non-preference-based measures have been designed for children/adolescents, such as the Pediatric Quality of Life Inventory (PedsQL) [20], which is a self-report questionnaire targeting children aged 8–12, and an adolescent version of the PedsQL that targets children/adolescents aged 13–18. Similarly, KIDSCREEN [21] was established in Europe and targets children/adolescents aged 8–18. Another measure, self-report version of the Child Health Questionnaire (CHQ), targets children/adolescents aged 5–18 [22].

Confirming the psychometric properties of HRQOL measures for younger people is particularly important, since they may lack the ability to comprehend fully the language and concepts used. This ability is influenced not only by age, but also social environment and language characteristics. As discussed above, the Japanese language uses a combination of two character systems. While children learn *hiragana* in the first grade, the numerous *kanji* are learned gradually over the course of their school careers. Although the Japanese version of the EQ-5D-Y limits the use of *kanji* to those learned during the first 2 years of elementary school (ages 6–8), the impact by the dual-character language system need to be confirmed. Moreover, no studies to date have compared self-report and proxy-report for the EQ-5D-Y to assess which of the two is more appropriate for properly capturing the health states of children/adolescents.

The EQ-5D for adults is used internationally as the de facto standard of PBMs [23]. The EQ-5D-Y uses a classification system similar to the EQ-5D, although the wording is tailored to children/adolescents. This study aimed to assess the psychometric properties of the Japanese version of the EQ-5D-Y within the context of the general Japanese population of children/adolescents. We expect that the EQ-5D-Y will be widely applied to the economic evaluation of healthcare technologies for younger people, as is the case for the EQ-5D among adults.

## Methods

### Instruments

The EQ-5D-Y comprises five items with three levels (no, some, and a lot): “mobility” (Item 1), “looking after myself” (Item 2), “doing usual activities” (Item 3),” having pain or discomfort” (Item 4), and “feeling worried, sad or unhappy” (Item 5). The words and phrases were modified to be more child-friendly while maintaining the domains of the adult version. The Japanese version of the EQ-5D-Y was prepared by a Japanese research group, which included the present authors, based on the first draft provided by the EuroQol group. The EuroQol group completed the process of translation, back translation, and harmonization, independently of the Japanese group. A linguistic pilot study with a small sample was performed by the Japanese group during the development process. Since the EQ-5D-Y targets children/adolescents aged ≥ 8, the Japanese version limits the use of *kanji* characters to those learned during the first two grades of elementary school (ages 6–8). In addition, each *kanji* character was provided with *furigana* (reading aid) using *hiragana*, to help children who have difficulty reading *kanji*. If predetermined value sets that reflect societal preferences of the general population are available, responses can be converted to an EQ-5D-Y index value. However, no value set for the EQ-5D-Y exists in any country yet, including Japan. Therefore, in the present survey, responses to the EQ-5D-Y were treated as ordinal variables.

The PedsQL Measurement Model is a generic profile-type measure of HRQOL for children/adolescents. The 23-item PedsQL Generic Core Scale consists of 23 items with five levels (never, almost never, sometimes, often, almost always) in four domains (physical functioning, 8 items; emotional functioning, 5 items; social functioning, 5 items; and school functioning, 5 items). Scores of each domain and total scores can be calculated based on responses to the questionnaire. The PedsQL has multiple versions that target different age groups. In the present study, two versions of the PedsQL were used for self-report, i.e., one for children aged 8–12 and the other for adolescents aged 13–15. A proxy version of the PedsQL has also been developed [24], and similarly has different versions based on age. Parents evaluated the health states of their children/adolescents using the appropriate proxy versions.

### Data collection

The survey was conducted in three major cities in Japan (Tokyo, Osaka, and Fukuoka) from February to March 2018. Different dialects are spoken in the three cities, but the Japanese version of the EQ-5D-Y with 3 levels uses the standard Japanese language (Tokyo dialect). Accordingly, in order to consider the influence of dialects, children/adolescents from two cities outside Tokyo were included. We targeted the general population of children/adolescents aged 8–15, which corresponds to the age group targeted by the original EQ-5D-Y.

Respondents were recruited by a research company (ANTERIO Inc.), which sampled more than 600 respondents in the three cities (i.e., roughly 200 respondents at each location) by non-random sampling. The sample number was not based on any rigid statistical considerations. Children/adolescents were stratified by sex and age. For the first-stage survey, after obtaining informed consent, parents (the father or mother) and their children/adolescents were asked to visit a specific location to answer the questionnaires to extend the commitment to take part in the survey. Children/adolescents responded to the EQ-5D-Y and PedsQL (using versions appropriate for their age) by self-report in a different room from that of their parents. Parents, in turn, were asked to evaluate their child’s health states using proxy versions of the EQ-5D-Y and PedsQL (using the proxy version appropriate for the age of the child/adolescent), as well as provide demographic information. We estimate that the surveys were completed within 30 min. For the second-stage survey, the only EQ-5D-Y was mailed to their residence after approximately 2 weeks, and both children/adolescents and their parents responded. We asked the same parents who responded to the first survey to cooperate in the second-stage survey. After completion, the response sheets were sent back to the authors for analysis.

This study was approved by the ethics committee of the National Institute of Public Health, to which the corresponding author belongs (NIPH-IBRA #12179).

### Reliability and construct validity

We principally followed the consensus-based standards for the selection of health measurement instruments (COSMIN) taxonomy for testing reliability and construct validity [25, 26]. Reliability was confirmed by self-report test–retest methods and comparison of responses by self-report and proxy-report by parents. As described above, retest was performed 2 weeks after the first-stage survey. We based the interval on the unlikelihood that the health states of children from the general population would change between the two time points; however, we cannot deny the possibility that the health states of some children/adolescents may have changed.

Regarding construct validity (also referred to as convergent validity), we compared responses to the EQ-5D-Y with responses to and scores of the PedsQL. The PedsQL is one of the most broadly used HRQOL measures for children/adolescents in Japan. In some studies that measured the construct validity of the EQ-5D-Y, the PedsQL was used [27, 28] together with other measures, such as KIDSCREEN. However, only one HRQOL measure was used considering feasibility of this survey.

### Statistical analysis

Summary statistics of background factors were calculated. The feasibility of the EQ-5D-Y was investigated by calculating the percentage of missing values. We calculated the percentage of worst-level responses because it is expected that few of the children/adolescents sampled from the general population actually have the worst-level state. Reliability was evaluated by calculating the percentage of agreement and kappa coefficients between (a) self-report test and retest responses, (b) self-report and proxy-report in the first-stage survey, and (c) proxy test and retest responses.

We interpreted kappa coefficients based on published criteria [29], as follows: kappa < 0.2 (poor agreement), 0.21–0.40 (fair agreement), 0.41–0.60 (moderate agreement), 0.61–0.80 (substantial agreement), and >0.80 (perfect agreement). Kappa coefficients were calculated for binary data (“no problem” and “any problems”). Since kappa coefficients have been reported to be easily influenced by prevalence, we used prevalence- and bias-adjusted kappa (PABAK) [30]. To consider factors that influence agreement, logistic regression (1: agreement, 0: disagreement) was performed by including certain background factors; inhabited area, sex, household income, number of parents, type of school. We also calculated intraclass correlation coefficients (ICCs) for visual analogue scale (VAS) scores.

Hypothesis testing for construct validity was investigated by Spearman’s rank correlation coefficients between responses to the EQ-5D-Y and PedsQL. We considered a correlation to be present when the absolute value of the correlation coefficient was > 0.3 (|*r*| > 0.3) [31]. Our hypothesis was that “mobility” of the EQ-5D-Y is correlated with the physical score of the PedsQL, and that ”having pain or discomfort” and “feeling worried, sad, or unhappy” of the EQ-5D-Y correlate with the emotional score of the PedsQL. We expected high correlation coefficients between the following items due to their similarities: (1) “mobility” of the EQ-5D-Y and the first (“It is hard for me to walk more than one block”) and second (“It is hard for me to run”) items in the physical domain of the PedsQL, (2) “looking after myself” of the EQ-5D-Y and the fifth item (“It is hard for me to take a bath or shower by myself”) of the physical domain of the PedsQL, (3) “doing usual activities” of the EQ-5D-Y and the first item (“I have trouble getting along with other kids”) of the social domain of the PedsQL, (4) ”having pain or discomfort” of the EQ-5D-Y and the seventh item (“I hurt or ache”) of the physical domain of the PedsQL, and (5) “feeling worried, sad, or unhappy” of the EQ-5D-Y and the second (“I feel sad or blue”) and fifth (“I worry about what will happen to me”) items of the emotional domain of the PedsQL.

## Results

A total of 654 children/adolescents responded to the questionnaires at the first-stage survey in three cities (219 each in Tokyo and Osaka, and 216 in Fukuoka). All participants (both children/adolescents and their parents) sent back their responses to the second-stage survey. Participant age and sex were well balanced. Participant characteristics are summarized in Table 1.

**Table 1:** Summary statistics of background characteristics

	Number	Percentage
	Or mean (SD)	
Region		
Tokyo	219	33.5
Osaka	219	33.5
Fukuoka	216	33.0
Age		
8	85	13.0
9	84	12.8
10	83	12.6
11	80	12.2
12	78	11.9
13	83	12.7
14	83	12.7
15	78	11.9
Gender		
Male	327	50.0
Female	327	50.0
Household income		
< JPY 5 millions	179	27.4
5 mil to < 10 millions	435	66.5
≥ 10 millions	40	6.1
Number of family members	3.3 (1.0)	
Number of siblings	1.2 (0.78)	
Accompanying parents		
Father	29	4.4
Mother	625	95.6
Number of parents		
1	65	9.9
2	589	90.1
School		
Elementary school	400	61.3
Junior high school	253	38.7
VAS score (self-report)	82.0 (17.1)	
VAS score (proxy-report)	88.3 (10.3)	
PedsQL total score (self-report)	89.5 (11.0)	

### Feasibility

All participants (both children/adolescents and their parents) responded to all items of the EQ-5D-Y for the first- and second-stage surveys. Similarly, all participants completed the VAS. There was one missing (self-report) response for the PedsQL. Four of 654 (0.6%) children/adolescents responded to Item 1 (“mobility”) with the worst-level response. Similarly, the numbers of children/adolescents who responded with worst-level responses were 0 (0%) for Item 2; 3 (0.5%) for Item 3; 27 (4.1%) for Item 4; and 22 (3.4%) for Item 5. On the other hand, the numbers of children who chose best-level responses were 495 (75.7%) for Item 1; 630 (96.3%) for Item 2; 593 (90.7%) for Item 3; 391 (59.8%) for Item 4; and 370 (56.6%) for Item 5. Only one child/adolescent had a VAS score of 0, and 23 (3.5%) had VAS scores < 50 points. In contrast, only two parents (0.3%) regarded their children’s health state as being < 50 points by VAS. The 25th percentile of VAS score was 74 (self-report) and 80 (proxy-report) points.

### Reliability

The mean period between responses to the first- and second-stage surveys was 16.9 days (SD: 2.2 days). The Chi-square test was performed to compare the distribution of EQ-5D-Y responses between self-report and proxy-report. This revealed significantly better evaluations by proxy-report compared to self-report. The percentage of agreement and Kappa coefficients (PABAK) of the EQ-5D-Y are provided in Table 2. The highest percentages of agreement and kappa coefficients were observed between proxy-report at the first- and second-stage surveys, whereas a comparison of self-report and proxy-report showed the lowest agreement. Items related to mental state (“having pain or discomfort” and “feeling worried, sad or unhappy”) had lower kappa coefficients among all three comparisons [(a) self- and self-report, (b) self- and proxy-report, and (c) proxy- and proxy-report], compared with the other three more physical items. In particular, kappa coefficients of mental items between self-report and proxy-report were less than 0.2, suggesting poor agreement. Kappa coefficients among all three comparisons for “mobility,” “looking after myself,” and “doing usual activities” were > 0.5 (fair to perfect agreement). “Looking after myself” had the highest kappa coefficient, at 0.9 (perfect agreement).

**Table 2:** Percentage of agreement and kappa coefficients: EQ-5D-Y

EQ-5D-Y	Comparison	Two categories		Three categories
		Percentage of agreement	PABAK	Percentage of agreement
Mobility	Self-report: first- versus second-stage survey	77.4	0.55	76.9
Looking after myself		95.9	0.95	95.9
Doing usual activities		89.1	0.78	88.8
Having pain or discomfort		69.7	0.39	67.3
Feeling worried, sad, or unhappy		65.1	0.30	62.8
Mobility	Self-report versus proxy-report at first-stage survey	74.9	0.50	74.8
Looking after myself		95.7	0.91	95.7
Doing usual activities		89.1	0.78	89.1
Having pain or discomfort		57.6	0.15	56.1
Feeling worried, sad, or unhappy		56.1	0.12	53.8
Mobility	Proxy-report: first- versus second-stage survey	92.7	0.85	92.4
Looking after myself		98.2	0.96	98.2
Doing usual activities		95.7	0.91	95.7
Having pain or discomfort		79.1	0.58	78.3
Feeling worried, sad, or unhappy		74.2	0.48	73.5

*PABAK* prevalence and bias-adjusted kappa

**Fig. 1:**
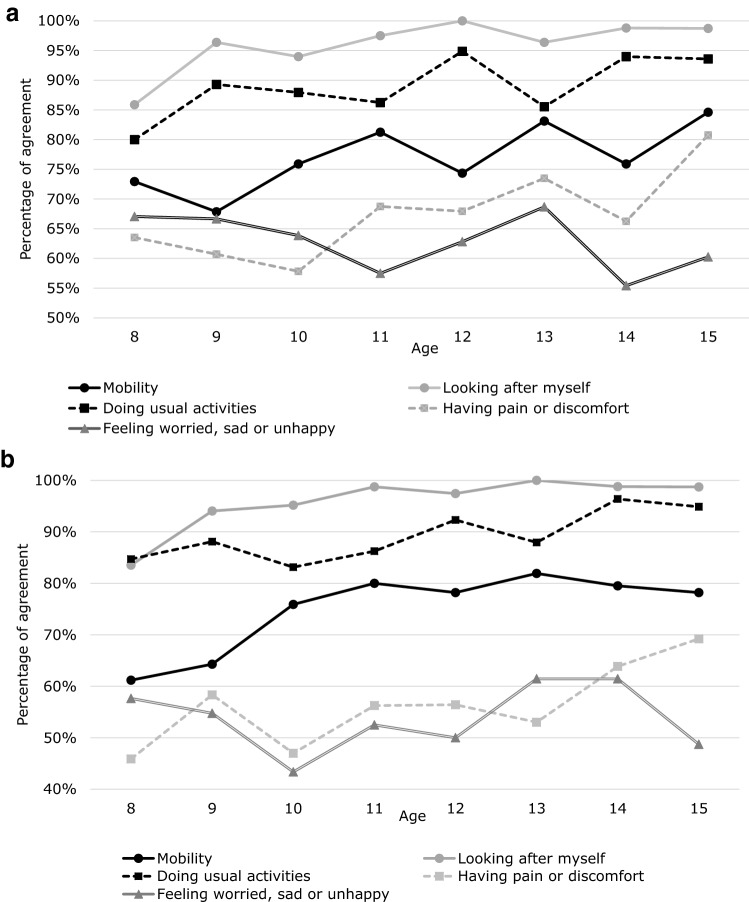
**a** Percentage of agreement (three categories) between self-report at first- and second-stage surveys. **b** Percentage of agreement (three categories) between self-report and proxy-report at first-stage survey

Figure 1 shows the percentage of agreement by age based on binary data (“no problem” and “any problems”). There is a tendency that the agreement between the pairs [(a) self- and self-report and (b) self-and proxy-report] is better for some items if the children/adolescents are getting older. Logistic regression confirmed the relationship between agreement of self-report responses at the two time points and background factors (Table 3), and relationship between agreement of self- and proxy-report responses and background factors (Table 4), and relationship between agreement of proxy-report responses at the two time points and background factors (Table 5). Almost all demographic factors were not significantly related to agreement, but agreement of some items is higher in older children/adolescents group (junior high school students) except Table 5. The relation seems stronger in the comparison of self-report and proxy-report. Percentage of agreement by subgroup is also shown in Tables 6, 7, and 8.

**Table 3:** Factors influencing agreement between self-report at first- and second-stage surveys

	Mobility		Looking after myself		Doing usual activities		Having pain or discomfort		Feeling worried, sad, or unhappy	
	Estimate	*P* value	Estimate	*P* value	Estimate	*P* value	Estimate	*P* value	Estimate	*P* value
City										
Tokyo	0.131	0.334	− 0.106	0.701	0.081	0.658	− 0.036	0.762	− 0.094	0.419
Osaka	− 0.123	0.349	0.167	0.564	− 0.135	0.435	− 0.005	0.963	0.194	0.099
Fukuoka	Reference									
Gender										
Female	− 0.045	0.628	− 0.314	0.122	0.077	0.540	− 0.096	0.255	0.099	0.224
Male	Reference									
Household income										
< JPY 5 millions	0.113	0.538	− 0.083	0.818	− 0.291	0.226	0.026	0.878	0.001	0.993
5 millions to < 10 millions	0.019	0.903	0.196	0.543	0.148	0.502	− 0.154	0.300	− 0.311	0.034*
≥ 10 millions	Reference									
School										
Elementary school	− 0.179	0.069	− 0.559	0.026*	− 0.176	0.187	− 0.210	0.017*	0.055	0.507
Junior high school	Reference									
Number of parents										
1	− 0.019	0.909	0.010	0.975	0.312	0.211	0.021	0.887	− 0.186	0.182
2	Reference									

*P < 0.05

**Table 4:** Factors influencing agreement between self−report and proxy-report

	Mobility		Looking after myself		Doing usual activities		Having pain or discomfort		Feeling worried, sad, or unhappy	
	Estimate	*P* value	Estimate	*P* value	Estimate	*P* value	Estimate	*P* value	Estimate	*P* value
City										
Tokyo	0.155	0.244	− 0.123	0.659	0.098	0.607	− 0.155	0.172	− 0.004	0.970
Osaka	− 0.152	0.236	− 0.194	0.479	− 0.160	0.366	0.013	0.907	− 0.076	0.499
Fukuoka	Reference									
Gender										
Female	− 0.038	0.680	− 0.408	0.051	− 0.112	0.383	− 0.098	0.220	0.108	0.173
Male	Reference									
Household income										
< JPY 5 millions	0.326	0.059	− 0.010	0.978	− 0.448	0.059	− 0.107	0.509	− 0.117	0.458
5 millions to < 10 million	0.240	0.101	0.250	0.443	0.375	0.094	− 0.193	0.171	− 0.132	0.339
≥ 10 millions	Reference									
School										
Elementary school	− 0.254	0.009**	− 1.094	0.003**	− 0.307	0.030*	− 0.164	0.046*	− 0.077	0.345
Junior high school	Reference									
Number of parents										
1	− 0.065	0.680	− 0.007	0.984	0.007	0.974	− 0.168	0.219	− 0.070	0.610
2	Reference									

*P < 0.05, **P < 0.01

**Table 5:** Factors influencing agreement between proxy-report at first- and second-stage surveys

	Mobility		Looking after myself		Doing usual activities		Having pain or discomfort		Feeling worried, sad, or unhappy	
	Estimate	*P* value	Estimate	*P* value	Estimate	*P* value	Estimate	*P* value	Estimate	*P* value
City										
Tokyo	0.104	0.633	− 0.558	0.180	0.268	0.375	− 0.020	0.882	− 0.042	0.740
Osaka	− 0.040	0.848	− 0.112	0.801	− 0.288	0.278	− 0.055	0.685	− 0.081	0.522
Fukuoka	Reference									
Gender										
Female	− 0.093	0.530	− 0.360	0.247	0.150	0.443	0.137	0.153	0.005	0.954
Male	Reference									
Household income										
< JPY 5 millions	0.105	0.689	0.059	0.913	0.031	0.932	0.167	0.357	0.090	0.603
5 millions to < 10 millions	0.317	0.161	0.272	0.562	0.150	0.640	0.126	0.415	0.057	0.702
≥ 10 millions	Reference									
School										
Elementary school	− 0.067	0.662	− 0.330	0.329	− 0.240	0.261	− 0.060	0.542	− 0.086	0.348
Junior high school	Reference									
Number of parents										
1	− 0.184	0.418	− 0.576	0.112	− 0.204	0.489	0.142	0.427	− 0.010	0.950
2	Reference

**Table 6:** Percentage of agreement between self-report at first- and second-stage surveys by each subgroup

	Mobility (%)	Looking after myself (%)	Doing usual activities (%)	Having pain or discomfort (%)	Feeling worried, sad, or unhappy (%)
City					
Tokyo	79.0	95.4	90.0	66.2	60.3
Osaka	74.9	96.3	87.2	67.1	67.1
Fukuoka	76.9	95.8	89.4	68.5	61.1
Gender					
Female	76.1	94.5	89.6	65.1	64.8
Male	77.7	97.2	88.1	69.4	60.9
Household income					
< JPY 5 millions	77.7	95.0	86.0	69.8	66.5
5 millions to < 10 millions	76.8	96.3	89.9	65.7	60.5
≥ 10 millions	75.0	95.0	90.0	72.5	72.5
School					
Elementary school	74.5	94.5	87.5	63.8	63.8
Junior high school	80.6	98.0	90.9	72.7	61.3
Number of parents					
1	76.9	95.4	92.3	69.2	56.9
2	76.9	95.9	88.5	67.1	63.5

**Table 7:** Percentage of agreement between self-report and proxy-report by each subgroup

	Mobility (%)	Looking after myself (%)	Doing usual activities (%)	Having pain or discomfort (%)	Feeling worried, sad, or unhappy (%)
City					
Tokyo	77.2	95.4	90.9	52.5	53.9
Osaka	72.6	95.0	87.2	56.2	51.6
Fukuoka	74.5	96.8	89.4	59.7	56.0
Gender					
Female	74.3	94.2	88.1	53.5	56.3
Male	75.2	97.2	90.2	58.7	51.4
Household income					
< JPY 5 millions	76.0	95.0	82.7	56.4	53.1
5 millions to < 10 millions	75.6	96.1	91.7	54.9	53.3
≥ 10 millions	60.0	95.0	90.0	67.5	62.5
School					
Elementary school	71.3	93.5	87.0	53.0	52.3
Junior high school	80.2	99.2	92.5	60.9	56.1
Number of parents					
1	73.8	95.4	86.2	49.2	50.8
2	74.9	95.8	89.5	56.9	54.2

**Table 8:** Percentage of agreement between proxy-report at first- and second-stage surveys by each subgroup

	Mobility (%)	Looking after myself (%)	Doing usual activities (%)	Having pain or discomfort (%)	Feeling worried, sad, or unhappy (%)
City					
Tokyo	93.2	97.3	96.8	77.6	72.6
Osaka	92.2	98.2	94.5	77.6	72.1
Fukuoka	91.7	99.1	95.8	79.6	75.9
Gender					
Female	91.7	97.6	96.3	80.7	75.8
Male	93.0	98.8	95.1	75.8	73.4
Household income					
< JPY 5 millions	91.1	97.8	95.0	79.9	74.3
5 millions to < 10 millions	93.3	98.4	96.1	78.4	73.4
≥ 10 millions	87.5	97.5	95.0	70.0	50.0
School					
Elementary school	92.0	97.8	95.0	77.5	72.3
Junior high school	92.9	98.8	96.8	79.4	75.5
Number of parents					
1	89.2	95.4	93.8	83.1	73.8
2	92.7	98.5	95.9	77.8	73.5

Mean scores of VAS were 82.0 (*N* = 654, SD: 17.1) by self-report in the first-stage survey, 88.3 (*N *= 654, SD: 10.3) by proxy-report in the first-stage survey, 85.4 (*N *= 654, SD: 16.8) by self-report in the second-stage survey, and 88.9 (*N *= 654, SD: 12.1) by proxy-report in the second-stage survey. VAS scores by proxy-report were significantly higher than scores by self-report (paired *t* test; *P* < 0.0001). Moreover, variance in VAS scores by proxy-report was smaller than that by self-report (*F* test; *P* < 0.0001). ICCs of VAS scores were as follows: 0.40 between self-report at the first- and second-stage surveys, 0.06 between self-report and proxy-report at the first-stage survey, and 0.31 between proxy-report at the first- and second-stage surveys. Agreement of VAS scores between self-report and proxy-report tended to be poor.

### Construct validity

Table 9 shows the correlation matrix between EQ-5D-Y responses and PedsQL scores by self-report in the first-stage survey. Item 1 of the EQ-5D-Y was correlated with the physical score of the PedsQL, but none of the mental scores. Similarly, Item 5 of the EQ-5D-Y was correlated with the emotional score of the PedsQL, but not the physical score. Item 4 of the EQ-5D-Y had correlation coefficients > 0.3 with physical, emotional, and social scores of the PedsQL.

**Table 9:** Correlations between EQ-5D-Y and PedsQL scores

PedsQL	EQ-5D-Y				
	Mobility	Looking after myself	Doing usual activities	Having pain or discomfort	Feeling worried, sad, or unhappy
Physical	0.38^a^	0.22	0.28	0.36^a^	0.23
Emotional	0.25	0.15	0.24	0.39^a^	0.39^a^
Social	0.26	0.18	0.27	0.31^a^	0.26
School	0.26	0.15	0.23	0.28	0.26
Total	0.35^a^	0.20	0.29	0.41^a^	0.36^a^

^a^Correlation coefficient > 0.30

Table 10 shows the correlation matrix between self-report responses to the EQ-5D-Y and PedsQL. Consistent with the hypotheses described in the *Statistical analysis* section, all correlation coefficients were > 0.3 (|*r*| > 0.3), except for (c) “doing usual activities” and the first item of the social domain of the PedsQL. No correlation was observed between proxy-report for the EQ-5D-Y and self-report for the PedsQL (Table 11). None of the coefficients exceeded our criteria. Proxy-report for the EQ-5D-Y had a lower construct validity than self-report for the EQ-5D-Y.

**Table 10:** Correlations between EQ-5D-Y by self-report and PedsQL by self-report responses

PedsQL	EQ-5D-Y				
	Mobility	Looking after myself	Doing usual activities	Having pain or discomfort	Feeling worried, sad, or unhappy
Physical					
Item 1	0.37^a^	0.24	0.20	0.16	0.05
Item 2	0.31^a^	0.14	0.22	0.19	0.15
Item 3	0.28	0.12	0.21	0.16	0.10
Item 4	0.21	0.11	0.11	0.15	0.12
Item 5	0.17	0.34^a^	0.07	0.11	0.06
Item 6	0.24	0.20	0.15	0.11	0.00
Item 7	0.17	0.16	0.16	0.41^a^	0.21
Item 8	0.27	0.18	0.23	0.30^a^	0.29
Emotional					
Item 1	0.22	0.16	0.20	0.33^a^	0.31^a^
Item 2	0.16	0.10	0.18	0.32^a^	0.33^a^
Item 3	0.18	0.10	0.14	0.30^a^	0.26
Item 4	0.14	0.12	0.14	0.25	0.19
Item 5	0.19	0.09	0.23	0.25	0.32^a^
Social					
Item 1	0.20	0.08	0.27	0.17	0.16
Item 2	0.17	0.10	0.19	0.17	0.13
Item 3	0.17	0.16	0.12	0.26	0.20
Item 4	0.20	0.16	0.11	0.22	0.16
Item 5	0.22	0.08	0.23	0.20	0.17
School					
Item 1	0.26	0.16	0.20	0.16	0.09
Item 2	0.18	0.07	0.16	0.15	0.23
Item 3	0.18	0.14	0.25	0.17	0.16
Item 4	0.13	0.09	0.05	0.22	0.19
Item 5	0.19	0.14	0.11	0.25	0.19

^a^ Correlation coefficient > 0.30

**Table 11:** Correlations between responses to EQ-5D-Y by proxy and PedsQL by self-report

PedsQL	EQ-5D-Y				
	Mobility	Looking after myself	Doing usual activities	Having pain or discomfort	Feeling worried, sad, or unhappy
Physical					
Item 1	0.01	0.10	0.04	0.11	0.11
Item 2	0.05	0.08	0.06	0.12	0.11
Item 3	0.10	0.10	0.07	0.17	0.14
Item 4	0.01	0.02	0.03	0.03	0.04
Item 5	0.01	0.24	0.10	0.05	0.05
Item 6	0.02	0.07	0.02	− 0.05	− 0.04
Item 7	0.02	− 0.01	0.01	0.05	0.00
Item 8	0.09	0.00	0.05	0.05	0.03
Emotional					
Item 1	0.03	0.04	0.02	0.01	0.01
Item 2	0.01	0.03	0.07	0.08	0.10
Item 3	− 0.02	− 0.01	0.01	0.08	0.03
Item 4	0.02	0.01	0.04	0.01	0.01
Item 5	0.04	0.07	0.11	0.04	0.07
Social					
Item 1	0.07	0.00	0.03	0.10	0.07
Item 2	0.00	0.01	0.06	0.02	0.07
Item 3	− 0.01	0.03	0.11	0.03	0.05
Item 4	0.05	0.01	0.08	0.05	0.02
Item 5	0.05	0.03	0.08	0.11	0.10
School					
Item 1	0.02	0.07	0.13	0.06	0.05
Item 2	0.02	0.03	0.07	0.04	− 0.02
Item 3	0.08	0.03	0.15	0.09	0.07
Item 4	0.06	0.03	0.09	0.06	0.07
Item 5	− 0.01	0.02	0.03	0.05	0.08

## Discussion

In this study, we surveyed psychometric properties of the Japanese version of the EQ-5D-Y. Our results suggest the EQ-5D-Y by self-report was feasible for Japanese children/adolescents aged 8–15. In terms of reliability, test–retest agreement was sufficiently high. For some items of the EQ-5D-Y, reliability for junior high school students was higher than that for elementary school students. The relation seems stronger in the comparison of self-report and proxy-report. Parents can understand children/adolescents’ health states better as they are growing up.

Reliability based on the kappa coefficients of self-report and proxy-report was different for the physical items and mental items. The proxy-report test–retest reliability was higher than the self-report test–retest reliability. Younger children’s feeling is generally more easily changed. In some cases, younger children responded to each item influenced by non-health-related events. For example, according to our experience of interview, a young child said that he/she is sad because my mom scolded him/her. Another child told us that it is difficult for him/her to walk around about because he/she got tired from coming here. Considering lower reliability, we may need to use self-report EQ-5D-Y more deliberately, and interpret the results more carefully than EQ-5D for adults. Construct validity of the EQ-5D-Y by self-report was confirmed by comparisons with the PedsQL. No correlations were observed between responses to the EQ-5D-Y by proxy-report and PedsQL by self-report. Overall, the result suggests that proxy responses may not sufficiently capture the health states of children/adolescents.

Some studies have reported on the psychometric properties of the EQ-5D-3L and -5L for adults in the general population [32–38]. However, reports on properties of the EQ-5D-Y in the general population are limited [27, 28, 39]. As the psychometric validation of EQ-5D-Y in general population is difficult in implementation, the small samples of disease-specific populations were reported in previous EQ-5D-Y validity studies. This fact justifies the novelty of our study. In a study that assessed the reliability of the self-reported EQ-5D-Y at two time points (7–10 days interval) [27], percentages of agreement (two categories of ‘”no problem” and ”any problems”) were 91.5% (Item 1), 93.8% (Item 2), 82.9% (Item 3), 69.8% (Item), and 78.3% (Item 5) in Italy, and 99.4%, 99.7%, 97.5%, 86.2%, and 87.4%, respectively, in Spain. Our present findings suggest that agreement in Japan is lower than those of Italy and Spain, although a common feature was the low agreement for items related to emotion. The ICCs of EQ-5D-Y VAS were 0.82 in Italy and 0.83 in Spain. These ICCs are higher than the ICC in Japan determined in the present study. Compared to children/adolescents in Italy and Spain, fewer Japanese children/adolescents responded with “no problem” for each item. The percentages of “no problem” responses were 93.5% (Item 1), 95.7% (Item 2), 84.3% (Item 3), 61.0% (Item 4), and 61.0% (Item 5) in Italy, and 95.3%, 98.6%, 93.7%, 80.0%, and 76.9%, respectively, in Spain. This may be one reason for the lower percentage of agreement in Japan. The ceiling effect of the EQ-5D-3L is well known and the 5L version was developed to address this issue [40, 41]. In some instances, children/adolescents who feel that “no problem” is not entirely accurate, but feel better than the description of the next level, might rate their health state as being the first level (i.e., “no problem”). In other instances, they might instead rate their health state as the next level.

One strength of our survey was that we achieved a 100% collection rate in the second survey. Only one response had a missing value for the PedsQL, but there were no missing values for the EQ-5D-Y in both surveys. This may have reduced any bias caused by missing values. However, there are also some potential limitations worth noting. First, the survey environment differed between the first- and second-stage surveys. Specifically, the first-stage survey was performed in a meeting room that respondents visited, whereas the second-stage survey took the form of a mail survey. We cannot deny the possibility that the change in environment could have influenced the responses in some way. Second, we targeted children/adolescents from the general population, who are likely to be relatively healthy. Further research will be needed to confirm the psychometric properties of the EQ-5D-Y for children/adolescents in clinical settings. Third, an external anchor was not used for selecting children/adolescents with stable health conditions over the 2 weeks for test–retest reliability assessment. The effect of changes in health conditions may be included in test–retest reliability assessment. We also did not obtain information about the illness of children/adolescents. Finally, we used only one HRQOL measure to assess construct validity. Specifically, we used only the PedsQL, since it is one of the most widely used HRQOL measures in Japan. We adopted this approach after considering the burden of responding to multiple questionnaires, but additional comparisons with other measures, such as KIDSCREEN, may help further confirm the construct validity of the EQ-5D-Y.

In conclusion, our results demonstrate that EQ-5D-Y is a feasible measure for use with children/adolescents in Japan, with sufficient reliability and validity. Proxy-report to the EQ-5D-Y was more reliable than self-report, but no construct validity was observed.
